# Demonstration of viability and fertility and development of a molecular tool to identify YY supermales in a fish with both genotypic and environmental sex determination

**DOI:** 10.1002/ece3.4148

**Published:** 2018-07-06

**Authors:** Ricardo Shohei Hattori, Seiya Tashiro, Yan Zhang, Naoya Kakuta, Masashi Yokota, Carlos Augusto Strüssmann, Yoji Yamamoto

**Affiliations:** ^1^ Unidade de Pesquisa e Desenvolvimento de Campos do Jordão APTA/SAA Campos do Jordão Brazil; ^2^ Graduate School of Marine Science and Technology Tokyo University and Marine Science and Technology Tokyo Japan

**Keywords:** genotypic sex determination, sex reversal, temperature‐dependent sex determination, Y‐linked gene

## Abstract

The pejerrey possesses a genotypic sex determination system driven by the *amhy* gene and yet shows marked temperature‐dependent sex determination. Sex‐reversed XY females have been found in a naturally breeding population established in Lake Kasumigaura, Japan. These females could mate with normal XY males and generate YY “supermale” individuals that, if viable and fertile, would sire only genotypic male offspring. This study was conducted to verify the viability, gender, and fertility of YY pejerrey and to develop a molecular method for their identification. Production of YY fish was attempted by crossing a thermally sex‐reversed XY female and an XY male, and rearing the progeny until sexual maturation. To identify the presumable YY individuals, we first conducted a PCR analysis using *amhy*‐specific primers to screen only *amhy*‐positive (XY and YY) fish. This screening showed that 60.6% of the progeny was *amhy*‐positive, which suggested the presence of YY fish. We then conducted a second screening by qPCR in order to identify the individuals with two *amhy* copies in their genome. This screening revealed 13 individuals, all males, with values twice higher than the other 30 *amhy*‐positive fishes, suggesting they have a YY complement. This assumption as well as the viability, fertility, and “supermale” nature of these individuals was confirmed in progeny tests with XX females that yielded 100% *amhy*‐positive offspring. These results demonstrate that qPCR can obviate progeny test as a means to identify the genotypic sex and therefore may be useful for the survey of all three possible genotypes in wild populations.

## 1 INTRODUCTION

The pejerrey *Odontesthes bonariensis* has a unique sex‐determining system that shifts between temperature‐dependent (TSD) and genotypic (GSD) sex determination depending on the temperature larvae experience during the first weeks of life (Strüssmann, Cota, Phonlor, Higuchi, & Takashima, 1996; Yamamoto, Zhang, Sarida, Hattori, & Strüssmann, 2014). As in other silversides such as *Odontesthes hatcheri* (Hattori, Strüssmann, Fernandino, & Somoza, 2013; Hattori et al., 2012) and *Hypoatherina tsurugae* (Bej, Miyoshi, Hattori, Strüssmann, & Yamamoto, 2017), in this species, the Y‐linked anti‐Müllerian hormone gene (*amhy*) is a strong testis determinant at intermediate temperatures around 25°C, whereby there is a prevalence of GSD over TSD (Yamamoto et al., 2014). However, higher or lower temperatures can override this genetic predisposition and yield female‐to‐male or male‐to‐female sex‐reversed individuals, respectively, characterizing TSD. The percentage of sex reversal increases proportionally toward extreme temperatures until reaching monosex offspring (Strüssmann et al., 1996).

Sex reversal may occur in natural fish populations of GSD/TSD‐bearing species like pejerrey as a result of climatic events or exposure to endocrine‐disrupting chemicals and cause imbalances in sex ratios (Brown et al., 2015; Cotton & Wedekind, 2009; Strüssmann, Conover, Somoza, & Miranda, 2010). In fact, sex‐reversed pejerrey males and females have previously been detected in a wild population in Lake Kasumigaura (Yamamoto et al., 2014). Both types of sex reversal in pejerrey appear to be viable and fertile, as for example in this study, and could mate in the wild causing skews in genetic sex ratios such as from the mating of sex‐reversed XX males with normal XX females or sex‐reversed XY females with normal XY males. The latter cross may potentially yield also YY offspring, sometimes called “supermales” because they would yield all‐XY progenies when mating with normal XX females (Cotton & Wedekind, 2009; Wedekind, 2012).

In order to estimate the possible impacts of climatic and anthropogenic factors on sex determination and, in turn, of genotypic/phenotypic sex imbalances on natural pejerrey resources, it is necessary to develop methods to assess the genotypic/phenotypic sex structure of wild populations. There is already a wealth of information on sex differentiation, reproduction, and molecular distinction of genetic males and females by amplification of the *amhy* gene in pejerrey (Strüssmann et al., 1996, 2010; Yamamoto et al., 2014), but information is completely lacking on the YY genotype. Although there are examples where YY fish are nonviable or infertile (*Betta splendens*, George, Pandian, & Kavumpurath, 1994; *Cichlasoma nigrofasciatum*, George & Pandian, 1996), many species can produce YY fish that are both viable and fertile (e.g. *Poecilia reticulata*, Kavumpurath & Pandian, 1992; *Oreochromis niloticus*, Mair, Abucay, Skibinski, Abella, & Beardmore, 1997; *Carassius auratus*, Yamamoto, 1975; *Salmo gairdneri*, Chevassus, Devaux, Chourrout, & Jalabert, 1988; *O. hatcheri*, Hattori, Oura, et al., 2009; see also Wedekind, 2012).

Given this background, we designed this study to assess whether YY pejerrey are viable, whether they develop as males or females, and whether they are fertile. We also developed a fast method to distinguish the YY from XY genotypes through DNA quantification of the Y chromosome‐linked *amhy* gene.

## 2 MATERIALS AND METHODS

### 2.1 Production of YY fish

Broodstock fish were procured from Yoshida Station, Field Science Center of Tokyo University of Marine Science and Technology (Shizuoka, Japan), where they are bred naturally. The effective population size is estimated in 500–1,000 fish, composed by phenotypic females and males at balanced proportions. The generation intervals of broodstock fish vary from 4 to 6 years. A sex‐reversed XY female discovered in a routine genetic screening of our broodstock and a normal XY male of pejerrey *O. bonariensis* were allowed to spawn naturally in a 1,000‐L recirculated tank with brackish water (0.2%–0.5% NaCl), under constant photoperiod (14‐hr light: 10‐hr dark) and temperature (17–18°C). The resulting progeny was reared at the same temperature from hatching until sexual maturity following conditions described in a previous study (Yamamoto et al., 2014). This temperature is expected to feminize part of the XY fish and was used on purpose to test if the YY genotype would yield higher rates of testicular formation than the XY. After 18 months, all remaining fish (*n* = 71) were anesthetized in buffered 2‐phenoxyethanol (Wako Pure Chemical Industries, Osaka, Japan) for the examination of gonadal sex by manual stripping of gametes/gonadal biopsy and for collection of fin clips for DNA analysis of genotypic sex. Previous to molecular sexing, fish were individually tagged and distinguished by subcutaneous injection of several combinations of colored latex ink (light green, pink, orange, and light blue; Environmental Technical Laboratory, Tokyo, Japan) in the dorsal flank area.

### 2.2 Screening of genetic males (XY/YY) through nonquantitative amhy amplification

To identify the presumable YY individuals, we first screened all fish (*n* = 71) with *amhy*‐specific primers to differentiate XX genotypes from XY and YY (Table 1). The extraction of genomic DNA from caudal fin and PCR analysis were performed following the conditions described in a previous study (Yamamoto et al., 2014). The *amhy*‐negative fish were transferred into a different tank, and the remaining *amhy*‐positive individuals were subjected to a second screening by quantitative PCR analysis to differentiate XY from YY genotypes as follows.

**Table 1:** List of primers, their respective sequences, and amplification conditions used for sex genotyping by quantification analysis (qPCR) of *amhy* gene

*Gene name* Primer ID	Sequence (5′–3′)	Amplification condition (two‐step cycle)
*amhy*: RT‐ObYYFw RT‐ObYYRv β*‐actin*:	TAGTTTCCYACCCCAGTC	95°C for 10 min, followed by 40 cycles of 95°C for 15 s and 60°C for 90 s
	CTGTTTTGTGATTTTCCGATGGGTT	
RT‐ObbactinFw RT‐ObbactinRv	GCTGTCCCTGTACGCCTCTGGCCTCTGG	
	GCTCGGCTGTGGTGGTGAAGC	

### 2.3 Screening of supermales (YY) through amhy quantification (qPCR)

The 5′ flanking region of *amhy* gene was used for designing the qPCR primers. This region was selected in order to avoid an eventual cross‐amplification with the *amha locus*, which shares high identity with the coding region of *amhy* gene (Yamamoto et al., 2014). The β*‐actin* gene was used as the positive control in all samples. All primer sequences and amplification conditions are listed in Table 2. Amplifications were performed with 50 ng of genomic DNA using SYBR Green Master Mix (Thermo Fisher Scientific, Waltham, MA) in a total reaction volume of 15 μl and quantification determined by the Standard Curve Method with four dilution points. All quantifications were performed in duplicate, and six XX individuals were included as negative controls.

**Table 2:** Results of progeny tests using *amhy*‐positive males classified as YY or XY by qPCR screening

Fish ID	Genotype based on qPCR	Number of fertilized embryos		
		XX	XY	Total
PR31	YY	0	24	24
PR36	YY	0	22	22
PR37	YY	0	12	12
PR39	YY	0	24	24
PR41	YY	0	16	16
PR42	YY	0	16	16
PR43	YY	0	15	15
PR01	XY	13	11	24
PR07	XY	14	10	24
PR15	XY	7	9	16
PR16	XY	5	6	11
PR20	XY	7	17	24

Oocytes from the same XX female were used in all crosses. No statistical difference from 1:1 was found between the frequency of XX and XY genotypes in crosses with presumable XY males (Chi‐square test, *p *< .05).

For distinguishing XY from YY genotypes, we performed a cluster analysis by the unweighted pair group method with arithmetic mean (UPGMA) using the average values of *amhy* for the two replicates of each individual after correction by the respective β*‐actin* values. We assumed that *amhy*/β*‐actin* ratios in YY would be twice as high as those of XY genotypes because the formers have theoretically two copies of the Y‐linked gene *amhy*. The differences (dissimilarity) between the *amhy*/β*‐actin* values for the genotypes were represented by the Euclidean distance. The UPGMA (group average method) cluster analysis was performed using R software, version 3.3.2 (R Development Core Team, 2011), based on the distance matrix of all individuals.

### 2.4 Confirmation of YY genotype by progeny test

Mature males identified as YY (*n* = 7) and XY (*n* = 5) in the molecular screening were selected for crossing with a single XX female. Crosses were performed by artificial insemination. Fertilized embryos were then incubated at 19°C for 1 week until reaching the eyed‐egg stage and then sampled (*n* = 11–24 embryos per cross) for *amhy* genotyping through *amhy* amplification as described for the first screening of parents. Fathers that generated all *amhy*‐positive progenies were scored as YY, whereas those that yielded progenies with both *amhy*‐negative and *amhy*‐positive were scored as XY. The deviations of genotypic sex ratios in the progeny tests were analyzed by the chi‐square test. Differences were considered as statistically significant at *p *<* *.05.

### 2.5 Test case of the molecular screening method

Screening of a broodstock population from the Yoshida Station, Field Science Center of Tokyo University of Marine Science and Technology (Shizuoka, Japan), was performed as a test case of the molecular identification of XY and YY genotypes. Populations in this propagation center are often reared at low temperatures and show a high frequency of male‐to‐female sex‐reversed individuals, increasing the probability of XY–XY crosses and the appearance of YY fish. As all YY obtained from the XY–XY cross in the first part of the study were males, this analysis was restricted only to phenotypic males. A total of 40 spermiating males identified by abdominal stripping were initially genotyped by simple PCR for the presence/absence of *amhy*. Subsequently, all *amhy*‐positive fish (*n* = 33) were genotyped by the quantification of *amhy* gene following the procedures described above.

## 3 RESULTS

### 3.1 Screening of XY/YY individuals through nonquantitative amhy amplification

**Figure 1:**
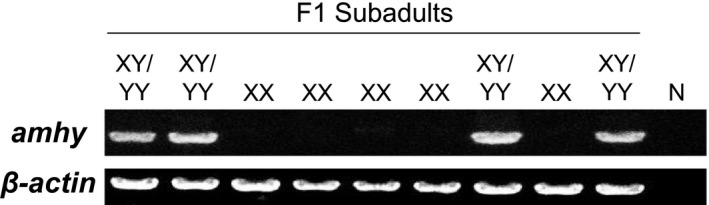
Sex genotyping by amplification of the *amhy* gene. Partial results of first screening conducted in nine F1 subadult individuals, which included genotypic females (XX) and genotypic males (XY/YY). β*‐actin* gene was used as positive control for all samples. N represents negative control (without DNA)

In the first screening, 28 (39.4%) of 71 subadult fish were genotyped as *amhy*‐negative (XX), whereas the remaining 43 (60.6%) as *amhy*‐positive. These *amhy*‐positive fish were assumed to include both XY and YY genotypes, as expected from an XY–XY cross (Figure 1).

### 3.2 Screening of YY individuals by amhy quantification

**Figure 2:**
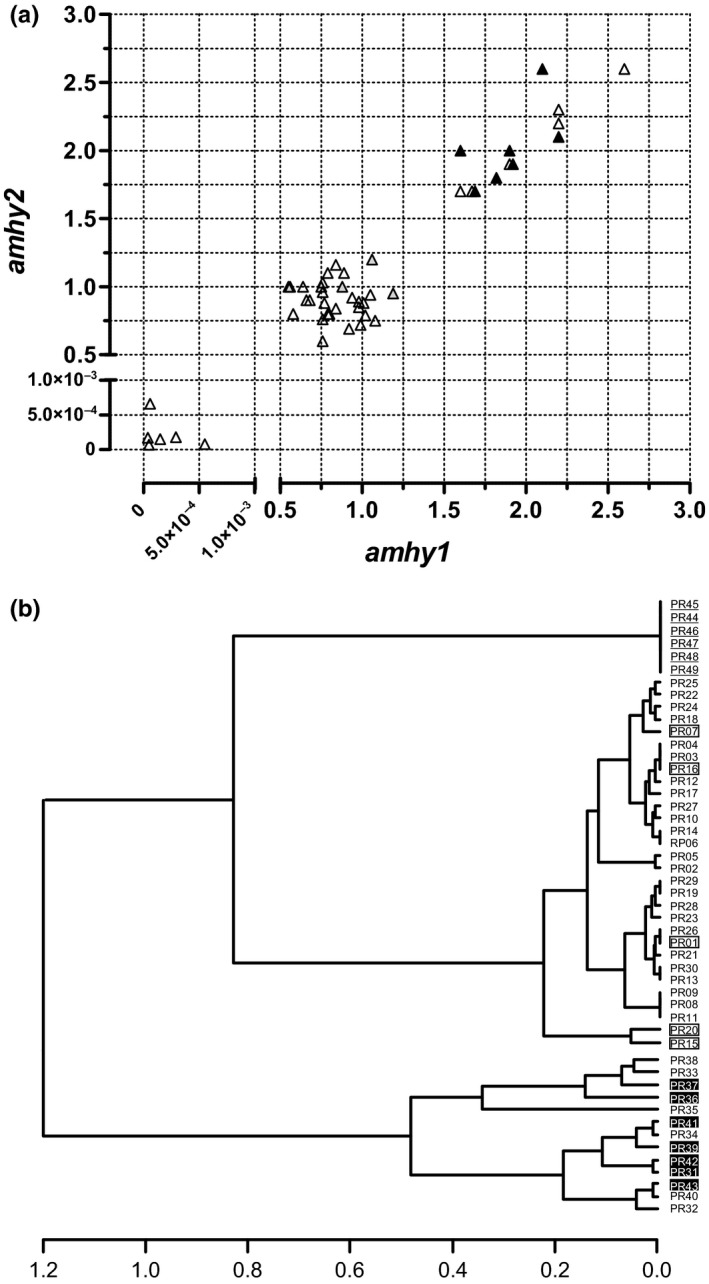
(a) Relationship between *amhy* replicate values (*amhy1* and *amhy2*) in the progeny (*n* = 50) of a cross of XY–XY parents. Black‐filled and gray‐filled triangles represent YY and XY genotypes, respectively, confirmed by subsequent progeny tests. Three patterns can be clearly visualized, whereby the individuals with low, intermediate, and high values represent the XX, XY, and YY groups, respectively. (b) Cluster analysis using the average values of *amhy* quantification for individuals shown in (a). Three main clusters were observed as follows: an upper cluster including only XX genotypes (ID numbers underlined), a middle cluster including five progeny‐tested XY fish (ID numbers in opened boxes) and other 25 individuals with intermediate *amhy* values, and a third cluster in the bottom including seven progeny‐tested YY fish (ID numbers in black‐filled boxes) and other six individuals with high *amhy* values

There was generally good agreement between replicate *amhy*/β*‐actin* ratios for each individual and the values grouped in three distinct clusters (Figure 2A). The UPGMA analysis confirmed these clusters and placed 30 of the *amhy*‐positive fish together with five progeny‐tested XY and the remaining 13 in another cluster with seven progeny‐tested YY individuals, whereas XX individuals grouped separately (Figure 2B).

### 3.3 Genotypic and phenotypic sex ratios

**Figure 3:**
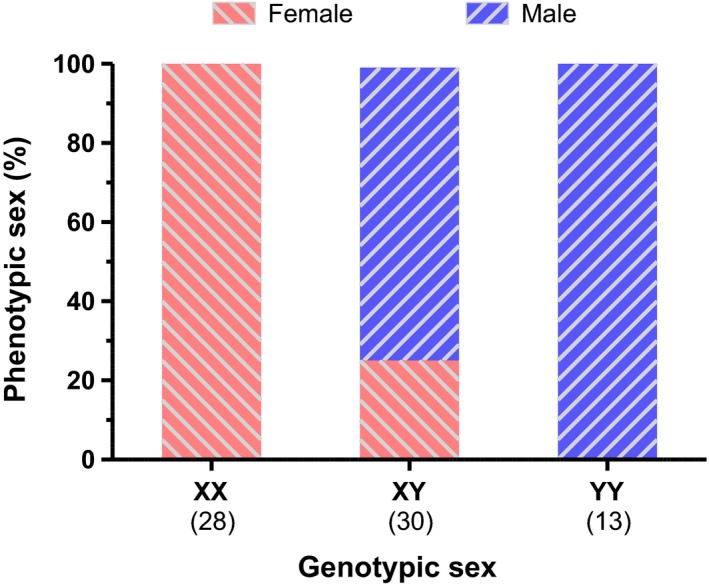
Histogram showing the relationship between genotypic and phenotypic sexes in the progeny of a cross of XY–XY parents. The genotypic sex of the fish was determined by qPCR and confirmed by progeny tests for seven YY and five XY fish. The number in parentheses represents the sample size

All fish identified as XX (*amhy*‐negative; 39.4%) in the molecular screening were phenotypic females, whereas the *amhy*‐positive (XY/YY) included both females and males. The YY individuals (18.3%) identified in the *amhy*‐quantification analysis were all phenotypic males, whereas the XY (42.3%) included 22 males and eight females, with a male‐to‐female sex reversal rate of 26.7% (Figure 3).

### 3.4 Confirmation of genotype by progeny test

**Figure 4:**
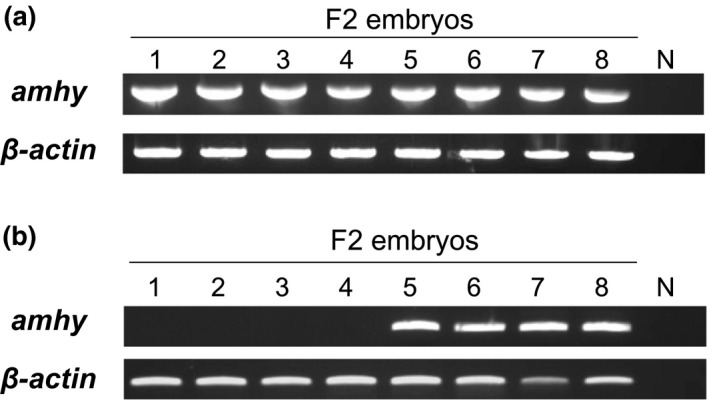
Representative results of the genetic screening of progenies from crosses between an *amhy*‐negative (XX) female and *amhy*‐positive males (XY/YY). The male in (a), genotyped as XY, generated a progeny with both *amhy*‐positive and *amhy*‐negative fish. The male in (b), genotyped as YY, generated an all *amhy*‐positive progeny. β*‐actin* was used as a positive control for all samples. Lanes 1–8 show the banding patterns for eight fish from each cross. N: negative control

The progenies derived from the crosses between an XX female and presumable YY fish were all *amhy*‐positive (Figure 4A; Table 2), whereas those derived from crosses of the same female with presumable XY fish showed both *amhy*‐negative and *amhy*‐positive genotypes in ratios that did not differ statistically from 1:1 (Figure 4B; Table 2).

### 3.5 Test case of the molecular screening method

**Figure 5:**
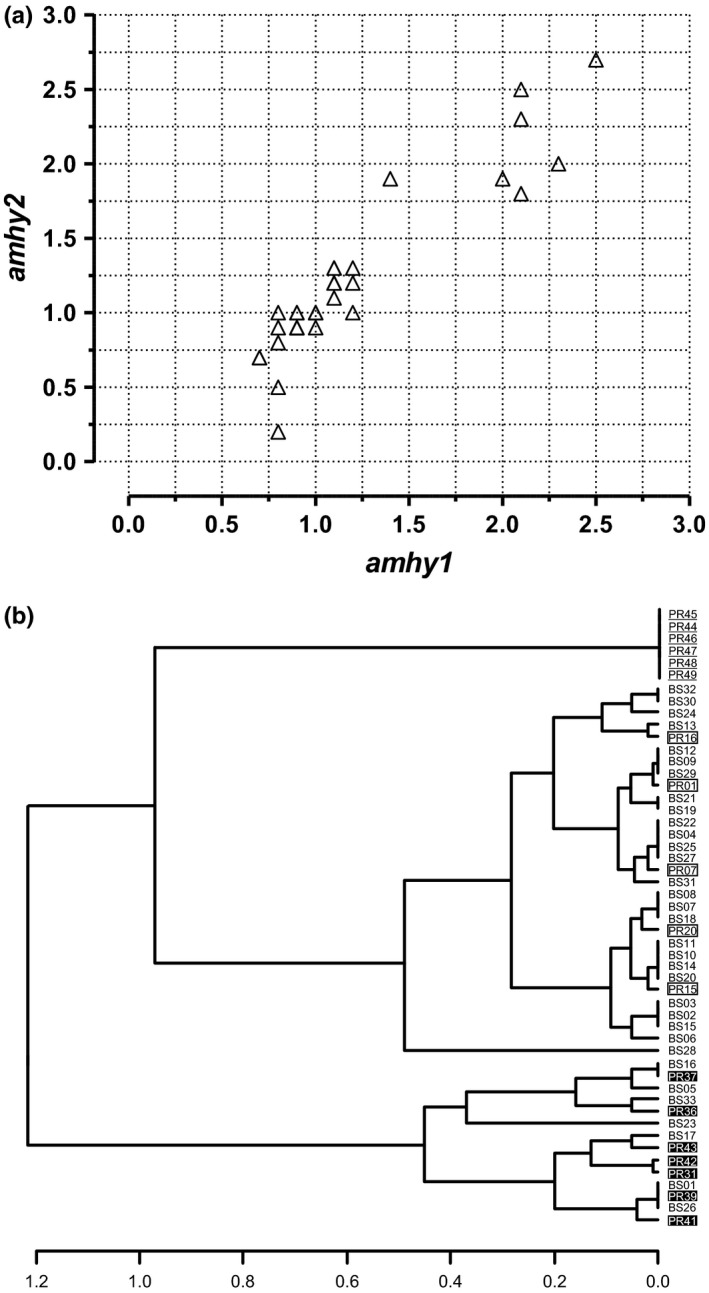
(a) Relationship between *amhy* replicate values (*amhy1* and *amhy2*) in the *amhy*‐positive male broodstock from Yoshida Station, Field Science Center of Tokyo University of Marine Science and Technology (*n* = 33). Two patterns can be clearly visualized, whereby the individuals with intermediate and high values represent the XY and YY groups, respectively. (b) Cluster analysis using the average values of *amhy* quantification for individuals shown in (a) and the same XX, XY, and YY fish shown in Figure 2a,b as reference samples. In b, three main clusters were observed as follows: an upper cluster including only XX genotypes (ID numbers underlined), a middle cluster including five progeny‐tested XY fish (ID numbers in opened boxes) and other 26 individuals with intermediate values, and a third cluster in the bottom with seven progeny‐tested YY fish (ID numbers in black‐filled box) and other seven individuals with high *amhy* values

The *amhy/*β*‐actin* ratios for *amhy*‐positive broodstock fish from Yoshida Station could be divided into two groups (Figure 5A). One group (*n* = 7) had high values similar to those of YY genotypes from the XY–XY cross while the other (*n* = 26) one had low values compared to those of the XY genotypes. In the UPGMA analysis, broodstock fish with high and low *amhy/*β*‐actin* ratios grouped together with progeny‐tested YY and XY fish, respectively (Figure 5B).

## 4 DISCUSSION

Temperature modulation of sex determination has been reported for many teleosts based on the results of laboratory experiments (Fernandino, Hattori, Acosta, Strüssmann, & Somoza, 2013; Strüssmann & Patiño, 1998), but temperature‐dependent sex reversal in the wild is still controversial. It has been suggested to occur in species of silversides of the genera *Menidia* and *Odontesthes* (Conover & Fleisher, 1986; Middaugh & Hemmer, 1987; Strüssmann et al., 2010) based on the observation of biased sex ratios, but a direct proof is not yet available. The discovery of the Y‐linked *amhy* gene in the pejerrey, an atherinopsid fish from South America, has made possible to screen for sex reversals in wild populations of this species, by distinguishing genotypic females from males and correlating them with gonadal sex (Yamamoto et al., 2014). However, due to the possible existence of YY individuals, a more precise method to distinguish all three genotypes (XX, XY, and YY) is needed. In this study, we confirmed the viability, sexual development, and fertility of YY pejerrey offspring from the mating of a sex‐reversed XY female and a normal XY male, and established a molecular sex genotyping method for identifying the three genotypes that could be used in wild‐caught samples.

The proportion of YY fish (*n* = 13) in relation to XY (*n* = 30) observed at the age of 18 months agreed with that based on Mendelian inheritance for the progeny of an XY–XY cross. As pejerrey males attain sexual maturity at about this age, we can therefore assume that the YY are as viable as the XY in this species at least up to the onset of reproduction in males. This has been observed also in many other teleosts, although in some species, YY are either not viable or infertile (reviewed by Devlin & Nagahama, 2002). The YY genotypes were also shown to be fertile and were all male even at the feminizing temperature of 17°C (Strüssmann et al., 1996). This contrasts with the fact that about one‐fourth of the XY fish differentiated as females and suggests that the presence of two *amhy* copies in the YY genotype makes the gonads less sensitive to feminization by low temperatures. We also noted while performing artificial fertilization that all 1‐year‐old YY males (*n* = 11) had abundant milt production compared to 36.4% (eight of 22) in XY males (unpublished observations), suggesting that the former may attain sexual maturation earlier than the latter. Studies including mRNA quantification of genes involved in *amh* signaling (such as *amhy*,* amha*, and *amhr2*) and measurement of androgens and stress hormones, which are related to sex determination in pejerrey (Fernandino, Hattori, Kishi, Strüssmann, & Somoza, 2012; Hattori, Fernandino, et al., 2009), will be conducted for comparison of thermal sensitivity between XY and YY fish. Similar studies should examine whether *amhy* has any role in the onset of puberty in this species that could explain the early maturation observed in YY fish.

A similar strategy of using qPCR from genomic DNA to distinguish YY and XY genotypes has been successfully employed to screen for YY supermales in brook trout *Salvelinus fontinalis* (Schill, Heindel, Campbell, Meyer, & Mamer, 2016) and in the vegetable crop *Asparagus officinalis* (Mutoh, Watanabe, Nii, & Kanno, 2009). In addition to being very fast, the qPCR showed 100% agreement with the results of genotypic sex inferred by progeny test, which demonstrates its reliability and potential usefulness for field studies. An alternative approach is the amplification of Y and X chromosome‐specific markers by nonquantitative PCR as has been used for distinction of YY supermales in yellow catfish *Pelteobagrus fulvidraco* (Wang, Mao, Chen, Liu, & Gui, 2009) and Nile tilapia *O. niloticus* (Li et al., 2015). However, in these cases, specific markers for both sex chromosomes are required, limiting the application of this method to a handful of species for which such markers have been developed. Genotyping by qPCR may allow screening not only of YY supermales but also of superfemales (WW in species with the ZZ‐ZW system) by quantification of a W‐linked genetic marker.

In addition to a test case screening of broodstock fish propagated in our field experimental station, we have recently started using the *amhy* qPCR method in field studies of natural pejerrey populations in Argentina. In both cases, we have uncovered YY supermales (preliminary results), suggesting that they may occur in the wild or in unassisted, naturally reproducing captive populations. In both locations, we also found male‐to‐female (XY females) sex reversals, probably as the result of low temperature‐induced feminization, and we suppose the YY fish were derived from mating of these XY sex‐reversed females with normal XY males. It has been shown that many areas around the globe are experiencing increases not only in average land and ocean temperature and frequency of heat waves, but paradoxically also in the frequency of cold outbreaks (IPCC, 2014; Peterson, Stott, & Herring, 2012). Our ongoing field studies should help clarify the causal relation of abnormal temperature fluctuations during the reproductive season and the probability of occurrence of feminization and masculinization in wild pejerrey populations. Considering that YY fish have been already found in natural populations, that they most likely develop as males, and that they are fertile, their effects on the XY chromosome balance of wild populations cannot be underestimated. In the worst scenario, the occasional occurrence of feminizing cold outbreaks (leading to the overabundance of the Y chromosome in the population through the formation of XY females and subsequently of YY supermales) interspersed with masculinizing warm conditions could lead to drastic masculinization and sudden demographic collapse (see e.g. Cotton & Wedekind, 2009).

In conclusion, these findings indicate that the presence of YY fish has to be considered in wild population surveys and that genotyping by qPCR might be an important tool for precise identification of all possible three sex genotypes (XX, XY, and YY). Together with studies on the effects of water temperature and endocrine‐disrupting chemicals (EDCs), simultaneous assessment of genetic and phenotypic sex can be invaluable for evaluating the effects of global climate change or anthropogenic impacts on reproductive health of fish populations, particularly in the case of species with similar sex determination mechanisms as pejerrey.

## CONFLICT OF INTEREST

Authors declare no conflict of interest.

## AUTHOR CONTRIBUTIONS

R.S.H., S.T., Y.Z., and N.K. conducted rearing experiments and genotyping analyses. Y.M. conducted data analysis. R.S.H., C.A.S., and Y.Y. wrote the manuscript.
